# Influence of Sleep Time on the Consumption of Ultra-Processed Foods in Adolescents in a Capital of Brazil: A Longitudinal Study

**DOI:** 10.3390/nu17010022

**Published:** 2024-12-25

**Authors:** Erika Mayra de Almeida Barreto, Aléssio Tony Cavalcanti Almeida, Adélia Costa Pereira Arruda Neta, Flávia Emília Leite de Lima Ferreira

**Affiliations:** 1 Programa de Pós-Graduação em Saúde Coletiva, Universidade Federal da Paraíba, João Pessoa 58051-900, Brazil; 2 Departamento de Economia, Universidade Federal da Paraíba, João Pessoa 58051-900, Brazil; alessiotony@gmail.com; 3 Programa de Pós-Graduação em Ciências da Nutrição, Universidade Federal da Paraíba, João Pessoa 58051-900, Brazil; adeliacpereira@gmail.com; 4 Departamento de Nutrição, Universidade Federal da Paraíba, João Pessoa 58051-900, Brazil; flaemilia@gmail.com

**Keywords:** adolescent, sleep habits, ultra-processed foods

## Abstract

Background/Objectives: The study prospectively assessed the association between sleep duration and the consumption of ultra-processed foods in adolescent students from a Brazilian capital. Methods: We observed 1431 adolescents participating in the Longitudinal Study on sedentary behavior, physical activity, dietary habits, and adolescent health, aged 10 to 14 years, in the years 2014 to 2017. The percentage of consumption of ultra-processed foods was evaluated by the Friedman test. Generalized Estimation Equation (GEE) models with parameters for robust analysis were used to verify the prospective association between sleep and the consumption of ultra-processed foods. Results: The percentage consumption of ultra-processed foods in relation to total daily calories increased in boys between 15 and 17 years old (31.8% in 2015 to 35.8% in 2017), being more stable among older girls (36.7% in 2015 to 37.7% in 2017), which may have occurred due to the influence of the social environment, reduced critical capacity, and lower concern for nutrition. This excessive consumption can lead to health and body composition impairments. Over time, adolescents who were in the fourth quartile of sleep had a 1.4% reduction (95% CI −2.49; −0.28) in the energy percentage from ultra-processed foods. When adjusting the initially used model in the GEE (Generalized Estimation Equation) with sociodemographic variables (income, maternal education, age) and lifestyle factors, sleep duration decreased by 2.1% (95% CI −4.09; −0.22) only in boys. The shorter duration of sleep in adolescents has been shown to be associated with unhealthy behavioral factors, such as a preference for fatty and sugary foods. There were no statistically significant associations for girls in this model. Conclusions: Sleep influences the consumption of ultra-processed foods in adolescents, showing that as sleep time increases, the consumption of foods classified as ultra-processed decreases.

## 1. Introduction

Sleep is a complex and essential biological *process*, a daily necessity for all human beings regardless of age, sex, or ethnic origin. In addition to maintaining the normal functioning of the brain, sleep plays an important role in controlling the functions of the other systems of the body and this becomes very evident in states of sleep deprivation [1]. Sleep disorders have affected individuals of all age groups, including adolescents, and can cause mental [2,3] and physical impairments, primarily impacting their metabolism. Ensuring an adequate sleep duration influences the production of growth hormones, essential for the development of muscles and bones. Sleep also plays a crucial role in the emotional development of adolescents, who are in a period of intense learning and socialization.

Restricted sleep time causes changes in the metabolism of lipids and glucose and reduces insulin sensitivity, in addition to causing a reduction in serum leptin, which is aggregated with increased satiety and the elevation of ghrelin. As a consequence of hormonal modification, there is an increase in appetite, which can trigger changes in the food choices of individuals, such as increased desire for foods of higher energy density [4,5,6].

The National Sleep Foundation [7] recommends that adolescents get eight to nine hours of sleep a day to avoid health problems. Approximately 70% of adolescents have an insufficient amount of sleep (<8 h/day) and 16.7% have a negative perception of sleep quality. In Brazilian adolescents, the negative perception of sleep quality has increased over 10 years, rising from 31.2% in 2001 to 45.9% in 2011. The decline in sleep quality may be influenced by the growing use of electronic devices among adolescents, study, or work schedules [8].

Sleep deprivation in adolescence can alter the amount, composition, and distribution of food intake, with the choice of ultra-processed foods being a detriment. Adolescents who sleep later are possibly more exposed to the consumption of unhealthy foods, and the time interval between the last meal at night and bedtime may be a determinant for the increase in caloric intake of this group [9,10,11]. A systematic review of sleep and eating patterns in children and adolescents showed that there is a direct relationship between healthier diets and adequate sleep duration and that children and adolescents who usually sleep later consume fewer fruits and vegetables and more “snacks”.

A study conducted with 14,400 children and adolescents, aged 6 to 18 years, concluded that short sleep duration is significantly associated with an increased chance of daily intake of snacks, soft drinks, fast foods, and beverages with added sugar. In addition, low sleep duration in students was associated with higher chances of daily consumption of sugary drinks, soft drinks, industrialized juices, milk, yogurt, and coffee [9]. In adolescence, individuals acquire autonomy for their food choices and the patterns established in this phase tend to persist into adulthood [11].

Most studies associating sleep duration with dietary intake in adolescents are cross-sectional, and to our knowledge, no longitudinal studies in Brazil have explored this association in adolescents. This study aimed to longitudinally analyze the relationship between sleep duration, overweight, and the consumption of ultra-processed foods in school-aged adolescents, utilizing data from the Longitudinal Study on Sedentary Behavior, Physical Activity, Eating Habits, and Adolescent Health (LONCAAFS).

## 2. Materials and Methods

### 2.1. Population and Sample

The baseline adolescents (2014) were in the sixth grade and could be between 10 and 14 years old, and they were followed for four years (2014 to 2017). The LONCAAFS Study (Longitudinal Study on Sedentary Behavior, Physical Activity, Dietary Habits, and Health of Adolescents) was conducted with a representative sample of adolescents aged 10 to 14 years in the base year, of both sexes, i.e., students in the sixth grade of Middle School from schools in the municipality of João Pessoa (PB), northeast of Brazil. The chosen age range in this study corresponds to the onset of adolescence, and through monitoring this population, it would be possible to observe changes in nutritional status, dietary habits, and sleep patterns that occur during this period between boys and girls.

To calculate the sample size, we considered a reference population size of 9520 adolescents; an outcome prevalence of 50%, as it is the maximum prevalence considered when data on the outcome are not available; a 95% confidence interval; an acceptable absolute error of 4 percentage points; and a design effect equal to 2. Epi Info 6.01 software was used to calculate the sample size (https://www.cdc.gov/epiinfo), accessed on 1 June 2014. The minimum sample size was set at 1130 adolescents, with an increase of 40% in order to compensate for losses and refusals. The calculated sample was 1582 adolescents, selected by single-stage clusters in 28 schools (14 municipal and 14 state) distributed proportionally according to size (number of students enrolled in the 6th grade) and geographic region in the municipality. All 6th-grade students were invited to participate in the study.

In 2014, the final sample consisted of 1431 adolescents. Between 2014 and 2015, approximately 17% of the sample was lost for follow-up, ending with 1182 adolescents in 2015. Between 2015 and 2016, there was a new loss of 21%, totaling 929, and between 2016 and 2017, 13% or 745 adolescents.

### 2.2. Data Collection

The data collection of the study occurred between February and December of each year. In each wave, the return to schools happened in the same period. Data collection was performed through face-to-face interviews, with the application of a questionnaire with an average duration of 50 min of interview, individually. The entire questionnaire was evaluated for its validity and reproducibility. For the present study, the variables selected were sociodemographic factors, class shift, sleep duration, sedentary behavior, BMI/age, food intake, and level of physical activity.

#### 2.2.1. Sociodemographic Variables

The sociodemographic variables were gender (male/female), age (in full years), skin color (brown, black, white, yellow, indigenous—categories proposed by IBGE, the Brazilian Institute of Geography and Statistics), mother’s education level (up to incomplete middle school, up to incomplete high school, completed high school, or further years), and economic class, according to the classification of the ABEP—Brazilian Association of Research Companies [12].

#### 2.2.2. Sleep Duration

Sleep was questioned from the times of sleep to waking up both on school days and weekends, with the following questions: “On a normal weekday (Monday to Friday) what time do you sleep? Wake up?”; “On a normal weekend day (Saturday or Sunday) what time do you sleep? Wake up?” The weighted average sleep time per day was calculated from the following equation: ((class nights × 5) + (weekend nights × 2)/7) [13]. Short sleep duration refers to less than 9 h of sleep per night [7].

#### 2.2.3. Dietary Intake

For the estimation of adolescents’ habitual food consumption, two 24 h dietary recalls were administered each year, with the first one covering the entire population and the second applied to 30% of the total sample. To reduce intrapersonal diet variability and enhance the accuracy of intake estimation, the Multiple Source Method (MSM) (https://msm.dife.de) accessed on 1 June 2017, was employed, considered the most suitable method for estimating repeated measures of consumption over a given period [14].

The procedure used for application was the Multiple Pass Method (MPM), composed of 5 steps, namely: (1) Quick listing of the foods recalled; (2) List of commonly forgotten foods; (3) Definition of the time and name of the meal; (4) Cycle of detailing and reviewing, where the details of consumption are questioned; and (5) Final Review, in search of important data not obtained [15].

Homemade measurements were obtained with the aid of photo albums, which contain images of standard home measurements and portions of foods that are difficult to measure [16].

#### 2.2.4. Anthropometric Nutritional Status

Objective measurements of height and weight were performed. Height was measured using a Sanny^®^ portable stadiometer, brand Sanny, manufactured in São Bernardo do Campo, São Paulo, Brazil, following the standardization described by Lohman, Roche, and Martorell (1992). To measure body mass, a Bioland^®^ digital scale was used, Bioland brand, manufactured in Piracicaba, São Paulo, Brazil, with a precision of 100 g, weighing the adolescent in an orthostatic position, facing the wall with the arms parallel to the body. The diagnosis of nutritional status was made based on BMI (body mass (kg)/height (m)^2^)/age, classified according to the criteria suggested by the WHO, according to age and sex [17]. All measurements were performed in duplicate, measuring a third time when the second measurement was different from the first.

#### 2.2.5. Level of Physical Activity

To measure the level of physical activity, we used the Physical Activity Questionnaire for adolescents (QAFA) [18,19], an instrument adapted from the Self-Administered Physical Activity Checklist and validated. At the time of data collection, the adolescents were encouraged to report whether they performed any physical activity in the week prior to the day of collection, and the duration of the event. The questionnaire contained 19 types of physical activities, with different levels of intensity ranging from moderate to vigorous, with active displacement, frequency, and duration of each activity. As a criterion for the classification of adolescents, the total time in minutes of the activities performed throughout the week was added, and those who totaled 420 min or more were classified as physically active.

#### 2.2.6. Sedentary Behavior

The variable sedentary behavior was obtained subjectively from questions regarding the performance of activities in the sitting/inclined position, such as watching television; watching DVDs; using a computer, cell phone, or tablet; and playing video games, considering the number of days and hours of use per day, during the week and weekends, in the week prior to the interview. To calculate the total time in sedentary behavior, the sum of the time spent in each equipment was calculated and the result was divided by seven to result in the average number of hours per day in sedentary behaviors. Sedentary behavior was defined as sitting/reclining, without lifting, ≥120 min/day [20].

### 2.3. Data Processing

The data were tabulated in the EpiData 3.1 program by double typing with automatic checking of the consistency of the answers through the tool “validate double entry” to identify typing errors, later corrected according to the values contained in the questionnaires.

The food and dietary intake data obtained through the R24h were processed in the *Virtual Nutri Plus^®^* 1.0 (www.virtualnutriplus.com.br, 1 June 2014) online software, accessed on 10 June 2018. After tabulation, the foods were categorized according to their degree of processing, based on the “NOVA classification”, with reference to the Food Guide for the Brazilian Population (2014), which subdivides foods into the following groups: in natura or minimally processed foods; culinary ingredients; processed foods and ultra-processed foods. Data were used only on the consumption of ultra-processed foods. The consumption of these foods was added for each individual in order to evaluate the percentage of caloric participation of ultra-processed foods in the total energy value (VET) daily consumption of adolescents. The values of interpersonal variability (estimation of the variance of habitual intake) were calculated, and the intrapersonal variability was removed (estimation of the variance of intrapersonal error) from the estimation of food intake by the Multiple Source Method (MSM—www.dife.de, 1 June 2014).

### 2.4. Statistical Analysis

Statistical data processing was performed using STATA software version 17.0. Descriptive statistics were used to characterize the variables. To evaluate the changes in the behavior of the variables over time in boys and girls, the ANOVA for repeated measures test was performed. When the variables did not fulfill the assumptions for the ANOVA calculation, the Friedman test was used. The percentage of consumption of ultra-processed foods was evaluated according to the characteristics of the sample using the Friedman test.

To test the longitudinal association between sleep duration and consumption of ultra-processed foods, the database was evaluated in a panel with unbalanced data, and the criterion for the individual’s permanence in the analysis was to have at least two measures of the main dependent and independent variables in the study. Sleep duration was considered the independent variable, being divided into quartiles, using the 1st quartile as a reference, and the percentage of consumption of ultra-processed foods (in relation to the total calories consumed by the individual) was considered the dependent variable. To estimate sleep duration per day, the adolescents reported the time they went to sleep and woke up on weekdays (Monday to Friday) and on the weekends (Saturday and Sunday). The average number of hours of sleep per day was calculated by multiplying the number of hours of sleep on weekdays by five and by two for weekend days, and then dividing the result by seven. Perception of sleep quality was assessed based on the question “In general, how do you assess the quality of your sleep?”, with the following response options: poor, normal, good, very good, and excellent. This assessment was carried out throughout the study, that is, annually for four years. Furthermore, for analysis purposes, this variable was recategorized as negative (bad and normal) and positive (good, very good, excellent) sleep quality. The questions regarding duration (ICC = 0.91) and sleep quality (Kappa = 0.59) obtained satisfactory levels of reproducibility.

Generalized Estimation Equation (GEE) models were used using the following robust analysis parameters: Gaussian family, due to the normal distribution of the dependent variable, and the identity link function. The family refers to the distribution of the dependent variable. Therefore, as our dependent variable has a normal distribution, the family chosen was Gaussian. As for the link function, this refers to a transformation of the dependent variable that allows estimation of the model. For our study, we chose the identity function, where f(x) = x. In this case, the dependent variable is not transformed. This binding can be used with any distribution.

The initial model shows the association of the percentage of energy of ultra-processed foods with sleep time. In the adjusted model, the variables were selected by the stepwise forward method. The final equation was adjusted for sex, age, income, mother’s education level, sedentary behavior, and physical activity. Adjustment variables were selected according to well-established associations with the outcome in the literature.

### 2.5. Ethical Aspects

The LONCAAFS project was submitted to and approved by the Ethics Committee on Research with Human Beings of the Health Sciences Center of the Federal University of Paraíba, with the certificate of Presentation for Ethical Appreciation CAAE: 15268213.0.0000.5188. Data collection for the LONCAAFS study was conducted only after obtaining consent from the State Secretariat of Education and Culture of Paraíba (SEEC/PB) and the Municipal Secretariat of Education and Culture (SEDEC/JP), followed by contact with the schools via phone call and scheduled visits. In each school, a meeting was held with the school administrators to present the project, providing the authorization letter and explanatory materials. During these visits, letters of consent and the ethical approval certificate were also provided. Subsequently, the study was presented to sixth-grade students, and the Informed Consent Form (ICF) was distributed to be signed by parents or guardians, as all participants were under 18 years old.

## 3. Results

Table 1 and Table 2 show the characteristics of boys and girls over time. It was observed that boys grew on average 18 cm in 4 years (1.50 m in 2014 and 1.68 m in 2017) and increased 13 kg in weight (44.63 kg in 2014 and 57.96 in 2017). The percentage of overweight and obese boys showed a significant drop (35.20 in 2014 to 21.87 in 2017). Sedentary behavior was high in all years for boys and girls, exceeding the recommendations of 120 min per day (final average of 192 min for boys and 169 for girls in 2017). Sleep time decreased by almost 1 h for boys gradually, from 9.7 h in 2014 to 8.8 in 2017 and by 2 h for girls (10 h in 2014 to 8.1 in 2017). The consumption of ultra-processed foods varied little between years, accounting for more than a third of the total daily calorie consumption for both sexes. All associations were statistically significant.

Table 3 and Table 4 show the percentage consumption of ultra-processed foods in relation to the total daily calories of boys and girls, according to sociodemographic characteristics. There were only statistically significant differences with the age variable, and it is worth mentioning that consumption seems to increase among the older boys (between 15 and 17 years old) (31.8% in 2015 to 35.8% in 2017) and is larger and more stable among older girls (36.7% in 2015 to 37.7% in 2017).

Table 5 presents the associations through the Generalized Estimation Equations of quartiles of sleep time with the percentage of energy from ultra-processed foods consumed by adolescents.

In model 1, the crude model shows the association between sleep time and the energy percentage of ultra-processed foods. Over time, adolescents in the fourth quartile of sleep had a reduction of 1.61% (95% CI −3.21; 0.00) for boys, and 1.62% (95% CI −3.11; −0.12) for girls, in the percentage of energy coming from ultra-processed foods. Model 2, adjusted by age, shows a decrease of 1.61% (95%CI (−3.21; 0.00) for boys and 1.60% (−3.09; −0.11) for girls in the energy percentage of consumption of ultra-processed foods in the fourth sleep quartile. In models 3 and 4, with the adjustment of sociodemographic variables (income, maternal education, age) and lifestyle, sleep time showed a decrease with the percentage of energy coming from ultra-processed foods of 2.1% (95% CI −4.09; −0.22) and 2.0% (95% CI −3.95; −0.10) only for boys, with no statistically significant association for girls.

## 4. Discussion

The present study showed that sleep duration has an influence on the consumption of ultra-processed foods in adolescents over time, especially in boys. It was observed that the longer the sleep time, the lower the intake of these foods. Girls progressively increased their consumption of ultra-processed foods over the 4 years and reduced their sleep time by almost 2 h, while boys fluctuated in this consumption and decreased their sleep time by about 1 1/2 h. The present study shows that for boys, sociodemographic and lifestyle factors influence this association. The presence of sex hormones near puberty, the increase in body fat stores, and differences in lifestyle can influence the consumption of ultra-processed foods [4].

Disorders caused by changes in sleep schedules increase appetite, decrease satiety, and modify food intake. Through hormonal disorders, we have the release of leptin, which is associated with the elevation of satiety, while ghrelin is a hormone that causes a hunger effect [4]. These hormonal modifications could be a possible explanation for the inverse proportionality between reduced sleep duration and increased consumption of ultra-processed foods in adolescents.

The population in our study had an average of adequate sleep in the first year (9 h), according to the NSF (National Sleep Foundation), and this decreased over the years. These findings are similar to those found in a cross-sectional study conducted with adolescents from all over Brazil, in which adolescents aged 15 years had an average sleep duration of 8 h/week [21]. However, the average number of hours of sleep in our population is much higher than in children and adolescents in other continents. Teenagers aged 12 to 18 in South Korea experience high academic stress during their studies. The average amount of time Korean teens devote to studies is around 7 h and 50 min a day; in contrast, Korean teens spend less time sleeping (7 h and 30 min a day) [3,22].

It was observed that a reduction in sleep hours, even if it seems modest, can cause changes in the consumption of ultra-processed foods, which in the long term may contribute to the incidence of chronic non-communicable diseases. The small beta values and larger standard errors may indicate that the true effect between exposure and outcome could be null, but in the last quartile we observed a result for boys that corroborates our hypothesis. Adolescents with longer sleep time had a 2% reduction in the energy consumption of ultra-processed foods over 4 years in our study. Through a modeling assessment to evaluate the projected impact of a modest change (3.7%) in energy consumption from ultra-processed foods in the adult population of Brazil, they observed a reduction of more than 36,000 deaths from cardiovascular diseases by 2048 [23,24].

The shorter duration and poorer quality of sleep in adolescents have been shown to be associated with less healthy behavioral factors such as higher intake of fatty and sugary foods [25,26,27,28,29]. However, the studies presented are cross-sectional, which makes it impossible to establish a causal relationship. A systematic review with a meta-analysis of cross-sectional studies in adolescents showed that there is an association between high consumption of ultra-processed foods and sleep duration [29]. The present study, of a longitudinal nature, reinforces the result presented by the other studies, showing this influence, especially in boys.

The fact that adolescents have the highest prevalence of excessive consumption of ultra-processed foods may be due to the influence of the social environment, lower critical capacity, and little concern with food and perception of body image [30,31].

In our study, from the models constructed, an influence of sociodemographic and lifestyle factors was observed in this association. With increasing age in adolescence, there is a progressive decrease in sleep hours, which seems to be shorter and of lower quality in adolescents with lower socioeconomic levels [32].

In Brazil and Latin America, this pattern is associated with lower income and lower levels of maternal education [33], a significant factor identified in the study population, as the selected children are members of families with low income and mothers with low educational attainment.

It is important to note that in Brazil, ultra-processed foods are cheaper than unprocessed or minimally processed foods. The adolescents interviewed in our study are from public schools, with the majority (75%) being in classes C and D. When observed, the ultra-processed foods most often consumed by them are packaged snacks and stuffed cookies, which have very affordable prices in the country.

Another important factor for the association between sleep time and the consumption of ultra-processed foods in the studied group was sedentary behavior. The time of sedentary behavior of adolescents was high over the years (≤120 min/day). In a study of adolescents, nearly half of adolescents with sedentary behavior greater than two hours a day consumed at least one ultra-processed food daily [34].

Adolescents with longer times of sedentary behavior are more exposed to advertisements that encourage the consumption of snacks and ultra-processed foods [6]. In another study, the influence of television media and its persuasion strategies influence the consumption of ultra-processed foods, reaching all classes and types of consumers [35].

The increased screen time may be tied to unhealthy food choices due to the marketing used by the industry. The practicality in the consumption and preparation of these foods, in addition to palatability and energy density, are preponderant factors in this choice. In this way, teenagers can ingest them while watching television, using the computer, or playing video games [36].

The high rate of sedentary behavior in adolescents may be associated with the lack of interventions aimed at reducing screen time, such as the creation of public policies capable of promoting activities for adolescents in the after-school shift. In Brazil, teaching is not full-time and adolescents study only one shift, with more free time to be in front of screens.

In addition to the marketing used by the industry to promote ultra-processed foods, some media messages revolve around freedom of choice and the promotion of relationships among adolescents, which can be an important factor in increasing the consumption of ultra-processed foods [37].

### Limitations of the Study

As a strength of the study, we can highlight the longitudinal model, which allowed us to follow the same population over four years. By following participants over time, the longitudinal study provided valuable insights into the evolution of phenomena, the identification of trends, and a deeper understanding of the relationships between variables. Many of the studies that evaluate the relationship between sleep duration and food consumption are cross-sectional in nature, which does not make it possible to establish a causal relationship. Another strength of the study was the evaluation of ultra-processed food consumption, obtained through two 24 h recalls applied each year, in order to observe the habitual consumption of the group, correcting for intra- and inter-individual variability.

As limitations, we followed adolescents only from public schools in the city, and these have a lower socioeconomic level, thus not allowing representation for all school adolescents. The data obtained for the evaluation of the participants’ sleep time were objective and collected through interviews and not by specific examination, such as polysomnography. However, most of the studies presented on this topic use this method to assess sleep duration. If the study had taken place in private schools, the socioeconomic profile of the students would have influenced the research results, as well as provided a more specific analysis of sleep duration.

## 5. Conclusions

According to the present study, sleep influenced the consumption of ultra-processed foods in adolescents, showing that as sleep time increases, the consumption of foods classified as ultra-processed decreases, an important factor in warning about the importance of adequate sleep time and a healthy lifestyle. Due to being a longitudinal study, capable of analyzing the same population at different time points, it was possible to assess students during a crucial period of their development, examining adolescent behavior and enabling the establishment of a causal relationship between sleep and dietary consumption.

## Figures and Tables

**Table 1:** Changes over time in the characteristics of male adolescent students (2014 to 2017) in the longitudinal study on Sedentary Behavior, Physical Activity, Eating Habits, and Adolescent Health (LONCAAFS) Brazil 2022 (N = 544).

Variables	2014	2015	2016	2017	
	Average	Average	Average	Average	*p*
	(SD)	(SD)	(SD)	(SD)	
Age	12.15	13.03	14.04	14.96	<0.001 *
	(0.98)	(0.92)	(0.90)	(0.88)	
Weight (kg)	44.63	49.74	53.93	57.96	<0.001 *
	(12.21)	(12.69)	(13.36)	(12.75)	
Height (m)	1.50	1.56	1.63	1.68	<0.001 *
	(0.09)	(0.08	(0.09)	(0.08)	
BMI (kg/m^2^)	19.68	20.11	20.23	20.40	<0.001 *
	(4.20)	(4.27)	(4.22)	(4.01)	
% overweight/obesity	35.20	33.17	26.85	21.87	0.51 **
Physical activity (min/day)	100.99	69.00	73.61	73.39	<0.001 *
	(75.99)	(58.88)	(63.87)	(60.53)	
Sedentary behavior (min/day)	255.44	236.17	230.75	192.66	<0.001 *
	(152.82)	(169.53)	(149.95)	(142.85)	
Sleep time (in hours)	9.73	9.60	9.24	8.85	<0.001 *
	(1.50)	(1.46)	(1.56)	(1.55)	
Consumption of ultra-processed products (%/day)	34.33	36.29	32.92	36.21	0.02 *
	(11.31)	(11.43)	(11.47)	(10.86)	

* Friedman test for *p*-value. ** ANOVA for repeated measurements test.

**Table 2:** Changes over time in the characteristics of female adolescents (2014 to 2017) in the Longitudinal Study on Sedentary Behavior, Physical Activity, Eating Habits, and Health of Adolescents (LONCAAFS). Brazil 2022 (N = 636).

Variables	2014	2017	2015	2016	
	Average	Average	Average	Average	*p* *
	(SD)	(SD)	(SD)	(SD)	
Age	11.84	13.03	13.81	14.72	<0.001
	(0.92)	(1.00)	(0.88)	(0.81)	
Weight (kg)	44.26	49.73	52.50	54.71	<0.001
	(10.41)	(12.23)	(11.32)	(11.82)	
Height (m)	1.50	1.56	1.57	1.59	<0.001
	(0.08)	(0.08)	(0.06)	(0.07)	
BMI (kg/m^2^)	19.61	20.45	21.15	21.52	<0.001
	(3.72)	(3.97)	(4.14)	(4.27)	
% overweight/obesity	31.22	32.41	30.69	27.46	
Physical activity (min/day)	66.94	65.71	46.67	40.83	<0.001
	(54.01)	(55.99)	(47.87)	(47.37)	
Sedentary behavior (min/day)	235.10	243.12	202.81	169.27	<0.001
	(154.19)	(164.28)	(145.37)	(153.15)	
Sleep time (in hours)	9.97	9.43	9.19	8.10	<0.001
	(1.52)	(1.60)	(1.67)	(1.77)	
Consumption of ultra-processed products (%/day)	36.76	36.12	37.08	39.76	0.11
	(11.77)	(11.40)	(12.34)	(11.53)	

* Friedman test for *p*-value.

**Table 3:** Percentage of total caloric intake from ultra-processed foods in males and sociodemographic variables, sleep, sedentary behavior, and practice of physical activity of the adolescent students studied (2014 to 2017) in the longitudinal study LONCAAFS. Brazil, 2022.

Variables	2014	2015	2016	2017	
	Average (SD)	Average (SD)	Average (SD)	Average (SD)	*p*
Age *					
10–11	34.5 (11.2)	33.6 (11.5)			<0.001
12–14	34.1 (11.5)	34.4 (11.3)	33.2 (11.2)	36.5 (10.5)	
15–17		31.0 (10.7)	31.3 (12.7)	35.8 (11.4)	
Mother’s schooling **					
Incomp. middle school	34.2 (11.3)	33.8 (10.4)	32.4 (10.7)	35.4 (12.1)	0.74
Incomp. high school	34.3 (11.5)	35.1 (10.9)	32.7 (11.5)	36.8 (11.1)	
Compl. high school or more	33.2 (10.8)	34.6 (11.9)	34.2 (11.8)	36.1 (10.6)	
Sleep time **					
≤9 h/d	35.0 (11.6)	34.3 (11.3)	32.7 (11.2)	36.8 (10.7)	0.70
≥9 h/d	34.0 (11.2)	34.1 (11.4)	33.2 (11.8)	35.5 (11.0)	
Physical Activity **					
Physically active	34.0 (11.3)	34.3 (11.2)	32.7 (11.3)	35.6 (11.0)	0.74
Physically inactive	35.0 (11.3)	34.1 (11.5)	33.1 (11.7)	36.8 (10.7)	
Sedentary behavior **					
<120 min/day	32.9 (11.2)	33.0 (12.1)	32.6 (11.3)	35.2 (11.5)	0.89
≥120 min/day	34.7 (11.3)	34.6 (11.0)	33.0 (11.6)	36.8 (10.5)	

* Friedman test. ** ANOVA for repeated measurements test.

**Table 4:** Percentage of total caloric intake from ultra-processed foods in females and sociodemographic variables, sleep, sedentary behavior, and practice of physical activity of the adolescent students studied (2014 to 2017) in the longitudinal study LONCAAFS. Brazil, 2022.

Variables	2014	2015	2016	2017	
	Average (SD)	Average (SD)	Average (SD)	Average (SD)	*p*
Age *					
10–11	37.2 (11.4)	37.9 (12.1)			<0.001
12–14	35.9 (12.4)	37.8 (11.3)	37.0 (12.4)	40.6 (11.7)	
15–17		36.7 (11.8)	37.7 (11.8)	37.7 (10.9)	
Mother’s schooling **					
Incomp. middle school	37.3 (12.3)	35.8 (10.9)	36.4 (11.1)	41.0 (11.2)	0.07
Incomp. high school	36.9 (10.6)	38.6 (12.3)	38.4 (13.5)	38.6 (13.2)	
Compl. high school or more	36.1 (12.3)	38.6 (10.8)	38.6 (1.4)	39.9 (10.4)	
Sleep time **					
<9 h/d	37.7 (12.6)	37.6 (10.9)	37.8 (12.7)	40.2 (11.7)	0.70
≥9 h/d	36.4 (11.5)	37.8 (11.8)	36.4 (12.0)	39.2 (11.3)	
Physical Activity **					
Physically active	36.8 (12.1)	37.9 (11.4)	36.2 (11.8)	38.9 (13.7)	0.13
Physically inactive	36.7 (11.5)	37.7 (11.5)	37.4 (12.5)	40.0 (10.9)	
Sedentary behavior **					
<120 min/day	35.6 (12.1)	38.1 (11.5)	37.1 (13.9)	39.0 (12.0)	0.73
≥120 min/day	37.2 (11.7)	37.7 (11.4)	37.1 (11.5)	40.4 (11.1)	

* Friedman test. ** ANOVA for repeated measurements test.

**Table 5:** Models of Generalized Estimation Equations (GEEs) between quartile of sleep and percentage of energy consumption of ultra-processed foods in participants of the longitudinal study on sedentary behavior, physical activity, eating habits, and health of adolescents (LONCAAFS). Brazil, 2023 (N = 1180).

Quartile Sleep Time	MODEL 1		MODEL 2		MODEL 3		MODEL 4	
	β		β		β		β	
	(95% CI)		(95% CI)		(95% CI)		(95% CI)	
	SE		SE		SE		SE	
	Boys	Girls	Boys	Girls	Boys	Girls	Boys	Girls
Quartile 1	Ref	Ref	Ref	Ref	Ref	Ref	Ref	Ref
Quartile 2	0.15	−0.29	0.18	−0.21	0.22	0.02	0.18	0.07
	(−1.27; 1.56)	(−1.73; 1.15)	(−1.24; 1.59)	(−1.65; 1.23)	(−1.48; 1.91)	(−1.70; 1.73)	(−1.51; 1.87)	(−1.65; 1.78)
	0.72	0.74	0.72	0.73	0.87	0.88	0.86	0.88
Quartile 3	0.11	−0.60	0.11	−0.59	0.14	−0.74	0.09	−0.72
	(−1.31; 1.53)	(−1.94; 0.73)	(−1.31; 1.53)	(−1.93; 0.74)	(−1.53; 1.80)	(−2.37; 0.89)	(−1.59; 1.77)	(−2.35; 0.91)
	0.72	0.68	0.73	0.68	0.85	0.83	0.86	0.83
Quartile 4	−1.61	−1.62	−1.61	−1.60	−2.15	−1.06	−2.03	−1.01
	(−3.21; 0.00)	(−3.11; −0.12)	(−3.21; 0.00)	(−3.09; −0.11)	(−4.09; −0.22)	(−2.83; 0.71)	(−3.95; −0.10)	(−2.78; 0.76)
	0.82	0.76	0.82	0.76	0.99	0.90	0.98	0.90

Percentage of ultra-processed energy (%): quartiles 2, 3, and 4 of sleep time distribution; 95%CI: 95% confidence interval; β: beta coefficient; SE: standard error. Model 1: sleep time and energy percentage of ultra-processed foods. Model 2: model 1 + age. Model 3: model 2 + sociodemographic factors (income, mother’s schooling). Model 4: model 3 + lifestyle (sedentary behavior, physical activity).

## Data Availability

Data are contained within the article.

## References

[1] De Oliveira J.P., Ramos R.V., de Sousa A.M., de Oliveira F.C.T., Baldoni N.R. (2021). Consequências nutricionais da privação de sono em crianças e adolescentes: Uma revisão integrativa. Res. Soc. Dev..

[2] Kaneita Y., Yokoyama E., Harano S., Tamaki T., Suzuki H., Munezawa T., Nakajima H., Asai T., Ohida T. (2009). Associations between sleep disturbance and mental health status: A longitudinal study of Japanese junior high school students. Sleep Med..

[3] Do Y.K., Shin E., Bautista M.A., Foo K. (2013). The associations between self-reported sleep duration and adolescent health outcomes: What is the role of time spent on Internet use?. Sleep Med..

[4] Durço M.S., Silva L.H.S.e., Mallet A.C.T., de Oliveira C.F. (2020). Obesidade, distúrbios do sono e qualidade de vida. Epistem. Transversalis.

[5] Gonçalves B., França V.F. (2021). Qualidade do sono de universitários: Associação com o estado nutricional e hábitos alimentares. Acta Elit. Salut..

[6] Santana M.O. Estratégias de Marketing na Publicidade Televisiva de Alimentos Ultraprocessados no Brasil. Repositorioufmgbr. https://repositorio.ufmg.br/handle/1843/34466.

[7] NSF–National Sleep Foundation (2015). National Sleep Foundation Recommends New Sleep Times. https://sleepfoundation.org/media-center/press-release/national-sleep-foundation-recommends-new-sleep-times.

[8] De Souza Neto J.M., da Costa F.F., Barbosa A.O., Prazeres Filho A., dos Santos E.V.O., de Farias Júnior J.C. (2021). Physical activity, screen time, nutritional status and sleep in adolescents in northeast braziL. Rev. Paul. Pediatr..

[9] Mozaffarian N., Heshmat R., Ataie-Jafari A., Motlagh M.E., Ziaodini H., Shafiee G., Taheri M., Mansourian M., Qorbani M., Kelishadi R. (2020). Association of sleep duration and snack consumption in children and adolescents: The CASPIAN-V study. Food Sci. Nutr..

[10] Garcez M.R., de Castro M.A., César C.L.G., Goldbaum M., Fisberg R.M. (2021). A chrononutrition perspective of diet quality and eating behaviors of Brazilian adolescents in associated with sleep duration. Chronobiol. Int..

[11] Alibabaei Z., Jazayeri S., Vafa M., Feizy Z., Sajadi Hezaveh Z. (2021). The association between dietary patterns and quality and duration of sleep in children and adolescents: A systematic review. Clin. Nutr. ESPEN.

[12] ABEP–Associação Brasileira de Empresas de Pesquisa. (Brasil) (2015). Critério de Classificação Econômica Brasil. www.abep.org.

[13] Mitchell J.A., Rodriguez D., Schmitz K.H., Audrain-McGovern J. (2013). Sleep duration and adolescent obesity. Pediatrics.

[14] Junior E.V., Fisberg R.M., Marchioni D. (2012). Is the food frequency consumption essential as covariate to estimate usual intake of episodically consumed foods?. Eur. J. Clin. Nutr..

[15] Moshfegh A.J., Rhodes D.G., Baer D.J., Murayi T., Clemens J.C., Rumpler W.V., Paul D.R., Sebastian R.S., Kuczynski K.J., Ingwersen L.A. (2008). The US Department of Agriculture Automated Multiple-Pass Method reduces bias in the collection of energy intakes. Am. J. Clin. Nutr..

[16] Zabotto C., Viana R., Gil M. (1996). Registro Fotográfico Para Inquéritos Dietéticos: Utensílios e Porções.

[17] BRASIL (2011). Ministério da Saúde. Secretaria de Atenção à Saúde. Departamento de Atenção Básica. Orientações para a Coleta e Análise de Dados Antropométricos em Serviços de Saúde: Norma Técnica do Sistema de Vigilância Alimentar e Nutricional-SISVAN/Ministério da Saúde, Secretaria de Atenção à Saúde.

[18] De Farias Júnior J.C., Lopes A.d.S., Mota J., Santos M.P., Ribeiro J.C., Hallal P.C. (2012). Validade e reprodutibilidade de um questionário para medida de atividade física em adolescentes: Uma adaptação do Self-Administered Physical Activity Checklist. Rev. Bras. Epidemiol..

[19] Filho A.P., Barbosa A.O., Mendonça G., Júnior J.C.D.F. (2017). Reproducibility and concurrent validity of the physical activity questionnaire for adolescents (QAFA) aged 10–14 years. Rev. Bras. Cineantropom. Desempenho Hum..

[20] Guerra P.H., de Farias Júnior J.C., Florindo A.A. (2016). Sedentary behavior in Brazilian children and adolescents: A systematic review. Rev. Saude Publica.

[21] Oliveira J.S., Barufaldi L.A., Abreu G.d.A., Leal V.S., Brunken G.S., Vasconcelos S.M.L., dos Santos M.M., Bloch K.V. (2016). ERICA: Use of screens and consumption of meals and snacks by Brazilian adolescents. Rev. Saúde Pública.

[22] Park S. (2014). Associations of physical activity with sleep satisfaction, perceived stress, and problematic Internet use in Korean adolescents. BMC Public Health.

[23] Moreira P.V.L., de Arruda A.D.C.P., de Lima Ferreira F.E.L., de Araújo J.M., da Costa Louzada M.L., de Lima R.L.F.C., de Toledo Vianna R.P., da Silva Neto J.M., Colombet Z., O’Flaherty M. (2022). Projected impact of change in the percentage of energy from each NOVA group intake on cardiovascular disease mortality in Brazil: A modelling study. BMJ Open.

[24] Franckle R.L., Falbe J., Gortmaker S., Ganter C., Taveras E.M., Land T., Davison K.K. (2015). Insufficient sleep among elementary and middle school students is linked with elevated soda consumption and other unhealthy dietary behaviors. Prev. Med..

[25] Ferranti R., Marventano S., Castellano S., Giogianni G., Nolfo F., Rametta S., Matalone M., Mistretta A. (2016). Sleep quality and duration is related with diet and obesity in young adolescent living in Sicily, Southern Italy. Sleep Sci..

[26] Oliveira G., da Silva I.B., de Oliveira ER A. (2019). O sono na adolescência e os fatores associados ao sono inadequado. Rev. Bras. Pesqui. Saúde/Braz. J. Health Res..

[27] Wang M., Zhong J.M., Hu R.Y., Gong W.W., Yu M. (2021). Sleep duration and behavioral correlates in middle and high school students: A cross-sectional study in Zhejiang province, China. Sleep Med..

[28] Bhurosy T., Thiagarajah K. (2019). Are Eating Habits Associated with Adequate Sleep among High School Students?. J. Sch. Health.

[29] Delpino F.M., Figueiredo L.M., Flores T.R., Silveira E.A., Silva Dos Santos F., Werneck A.O., Louzada M.L.D.C., Arcêncio R.A., Nunes B.P. (2023). Intake of ultra-processed foods and sleep-related outcomes: A systematic review and meta-analysis. Nutrition.

[30] Correia B.A. (2016). Determinantes do Consumo de Alimentos Processados e Ultraprocessados em Estudantes da Universidade de Brasília (UnB), Distrito Federal. Bdmunbbr. https://bdm.unb.br/handle/10483/16466.

[31] Silva J.B., Elias B.C., Warkentin S., Mais L.A., Konstantyner T. (2022). Factors associated with the consumption of ultra-processed food by Brazilian adolescents: National Survey of School Health, 2015. Rev. Paul. Pediatr..

[32] Felden É.P.G., Leite C.R., Rebelatto C.F., Andrade R.D., Beltrame T.S. (2015). Sleep in adolescents of different socioeconomic status: A systematic review. Rev. Paul. Pediatr..

[33] Sosso F.E., Khoury T. (2021). Socioeconomic status and sleep disturbances among pediatric population: A continental systematic review of empirical research. Sleep Sci..

[34] Costa C.d.S., Flores T.R., Wendt A., Neves R.G., Assunção M.C.F., Santos I.S. (2018). Comportamento sedentário e consumo de alimentos ultraprocessados entre adolescentes brasileiros: Pesquisa Nacional de Saúde do Escolar (PeNSE), 2015. Cad. Saúde Pública.

[35] Monteiro C.A., Cannon G., Moubarac J.C., Levy R.B., Louzada M.L.C., Jaime P.C. (2017). The UN Decade of Nutrition, the NOVA food classification and the trouble with ultra-processing. Public Health Nutr..

[36] De Oliveira L.M.F.T., da Silva A.O., dos Santos M.A.M., Ritti-Dias R.M., Diniz P.R.B. (2018). Exercício físico ou atividade física: Qual apresenta maior associação com a percepção da qualidade do sono de adolescentes?. Rev. Paul. Pediatr..

[37] Stangherlin L. (2020). Influência do Consumo de Alimentos Ultraprocessados e do Comportamento Sedentário no Excesso de Peso de Adolescentes em uma Escola do Ensino Privado de Criciúma-SC. http://repositorio.unesc.net/handle/1/7491.

